# Design, synthesis, docking study and cytotoxic activity evaluation of some novel letrozole analogs

**DOI:** 10.1186/s40199-014-0083-4

**Published:** 2014-12-24

**Authors:** Mohsen Vosooghi, Loghman Firoozpour, Abolfazl Rodaki, Mahboobeh Pordeli, Maliheh Safavi, Sussan K Ardestani, Armin Dadgar, Ali Asadipour, Mohammad Hassan Moshafi, Alireza Foroumadi

**Affiliations:** Department of Medicinal Chemistry, Faculty of Pharmacy, Tehran University of Medical Sciences, Tehran, Iran; Drug Design and Development Research Center, Tehran University of Medical Sciences, Tehran, Iran; Institute of Biochemistry and Biophysics, University of Tehran, PO Box 13145–1384, Tehran, Iran; Department of Biotechnology, Iranian Research Organization for Science and Technology, Tehran, Iran; Neuroscience Research Center, Institute of Neuropharmacology, Kerman University of Medicinal Sciences, Kerman, Iran; Pharmaceutical Sciences Research Center, Tehran University of Medical Sciences, Tehran, Iran

**Keywords:** Breast cancer, Non-steroidal aromatase inhibitor, Cytotoxic activity

## Abstract

**Background:**

Breast cancer is the most common type of female cancer. One class of hormonal therapy for breast cancer drugs -non steroidal aromatase inhibitors- are triazole analogues. In this work, some derivatives of these drugs was designed and synthesized. All synthesized compounds were evaluated for their cytotoxic activities on breast cancer cell lines (MDA-MB-231, T47D and MCF-7).

**Methods:**

Our synthetic route for designed compounds started from 4-bromotolunitrile which was reacted with 1*H*-1,2,4-triazole to afford 4-(4-cyanobenzyl)-1,2,4-triazole. The reaction of later compound with aromatic aldehydes led to formation of the designed compounds. Eleven novel derivatives 1a-k were tested for their cytotoxic activities on three human breast cancer cell lines.

**Results:**

Among the synthesized compound, 4-[2-(3-chlorophenyl)-1-(1*H*-1,2,4-triazol-1-yl)ethenyl]benzonitrile (**1c**) showed the highest activity against MCF-7 and MDA-MB-231 cell lines and 4-[2-(4-methoxyphenyl)-1-(1*H*-1,2,4-triazol-1-yl)ethenyl]benzonitrile (**1 h**) exhibited highest activity against T47D cell line. According to cytotoxic activities results, compound 4-[2-(4-dimethylamino)-1-(1*H*-1,2,4-triazol-1-yl)ethenyl]benzonitrile (**1 k**) showed comparative activity against T47D and MDA-MB-231 cell lines with compound (**1 h**) and our reference drug Etoposide.

**Conclusion:**

In the process of anti-cancer drug discovery, to find new potential anti-breast cancer agents, we designed and synthesized a novel series of letrozole analogs. Cytotoxicity evaluation revealed that compounds (**1c**) and (**1 k**) were the most potent compounds with comparative activity with Etoposide. The results revealed that π-π interactions are responsible for the enzyme inhibitions of compounds (**1 c**) and (**1 k**).

## Background

Breast cancer is the most common female cancer. According to the American cancer society’s report about 12% of women in the U.S. will develop some invasive breast cancer during their lifetime. However breast cancer treatment has a complicated process and problems, chemotherapy resistance, surgery and available anti-tumor drugs side effects make it more difficult to gain the appropriate treatment regimen; consequently, there is great demand to introduce new active compounds with more anticancer activity and less unwanted reaction [1,2].

There is some different type of systemic therapy for breast cancer, one kind is hormonal therapy. Hormonal therapy can be given to women whose breast cancers test positive for estrogen to lower estrogen levels. Letrozole is a third generation of non-steroidal aromatase inhibitor – one class of hormonal therapy drugs- that was first introduces by Novartis to the market as Femara® for the treatment of local or metastatic breast cancer [3-5].

**Figure 1:**
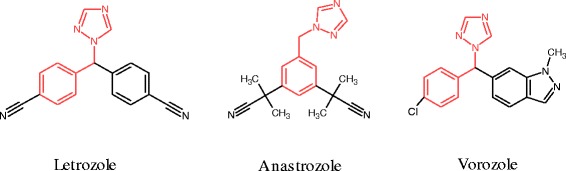
**Structure of non-steroidal aromatase inhibitors.**

Non-steroidal aromatase inhibitors (as shown in Figure 1) are triazole or imidazole analogues that bind to the active site of enzyme by coordinating the heme iron atom of active site through a heterocyclic nitrogen lone pair [5,6].

**Scheme 1:**
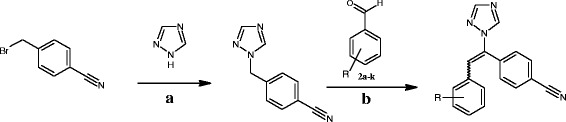
**The Synthesis rout of 4-[2-aryl-1-(1** ***H*** **-1,2,4-triazol-1-yl)ethenyl] benzonitriles 1a-k. (a)** MeOH, KOH, DMF, **(b)** 1,4-Dioxane, recrystallized in EtOH.

As it shown in Figure 1, 1-benzyl-1*H*-1,2,4-triazole scaffold is a conservative section of aromatase inhibitors which contains various moieties attached to the aliphatic carbon part of this scaffold. In continuation of our research program to find a novel anticancer agent [7-11], and considering the above mentioned data, in the current study, we report the synthesis of a novel series of substituted ethenylbenzene derivatives which linked to1-benzyl-1*H*-1,2,4-triazole (**1a-k**) and evaluated against three human breast cancer cell lines (Scheme 1).

## Methods

### Chemistry

All raw-materials, solvents and reagents were provided from Aldrich Chemicals and Merck AG. A Kofler hot stage apparatus was used for determination of melting points. The IR and ^1^HNMR Spectra were determined on a Shimadzu 470 (potassium bromide disks) and a Bruker 500 spectrophotometer respectively. Tetramethylsilane (TMS) was used as internal standard and chemical shifts are reported in ppm relative to it. The elemental analysis for C, H and N were taken by a Perkin-Elmer 843 spectrometer with using KBr as diluent. Electrospray ionization mass spectra (ESI-MS) were recorded by using Agilent 6410 Triple Quad. LC/MS.

Key intermediate 4-(4-cyanobenzyl)-1,2,4-triazole was prepared according to Doiron J. and his collogues report [12].

#### General procedure for preparing of 4[2-aryl-1-(1H-1, 2, 4-triazol-1-yl)ethenyl]benzonitrile (1a-k)

4-(4-Cyanobenzyl)-1,2,4-triazole (1Gr) and 1,4-Dioxane (10 mL) were added to the reaction vessel and stirred. Sodium hydride (0.27 Gr 60%) was added to the reaction mixture in 0–5°C and stirred for 30 minutes. Corresponding aldehyde (0.5 mmol) was added to the mixture and stirred at room temperature for 30 minutes. Ethanol (3 mL) was added to the reaction mixture at 60°C and stirred for an hour. Reaction mixture cooled to room temperature and mixture of ice-water (25 Gr) was added. Precipitate was filtered and recrystallized in ethanol to yield corresponding compound (**1a-k**).

#### 4-[2-Phenyl-1-(1H-1, 2, 4-triazol-1-yl)ethenyl]benzonitrile (1a)

Yield: 73%, mp 141–146°C. IR (KBr, cm^−1^) ν_max_: 2245 (nitrile), 1630 (C = C). ^1^H NMR (500 MHz, CDCl_3_): *δ* 8.25(s, 1H, triazole), 8.05 (s, 1H, triazole), 7.70-7.68 (d, *J* = 8.55 Hz,2H, benzonitrile), 7.35-7.33 (d, *J* = 8.55 Hz, 2H, benzonitrile), 7.30-7.26 (m, phenyl and ethenyl), 6.88-6.87 (d, *J =* 7.3 Hz, 1H, phenyl), ESI-Mass *m/z*: 272 [M]^+^.

#### 4-[2-(2-Chlorophenyl)-1-(1H-1,2,4-triazol-1-yl)ethenyl]benzonitrile (1b)

Yield: 70%, mp 141–144°C. IR (KBr, cm^−1^) ν_max_: 2240 (nitrile), 1622 (C = C).^ 1^H NMR (500 MHz, CDCl_3_): *δ* 8.16(s, 1H, triazole), 7.96 (s, 1H, triazole), 7.72-7.71 (d , *J* = 8.45 Hz, 2H, benzonitrile), 7.43 (s, 1H, ethenyl), 7.45 (d, *J* = 7.75 Hz, 1H, phenyl), 7.40-7.39 (d, *J* = 8.45 Hz, 2H, benzonitrile), 7.27-7.23 (t, *J* = 7.9 Hz, 1H, phenyl), 7.08-7.05 (t, J = *J* = 7.15 Hz, 1H, phenyl), 6.61-6.59 (d, *J* = 7.75 Hz, 1H, phenyl), ESI-Mass *m/z*: 306 [M]^+^.

#### 4-[2-(3-Chlorophenyl)-1-(1H-1,2,4-triazol-1-yl)ethenyl]benzonitrile (1c)

Yield: 74%, mp 137–140°C. IR (KBr, cm^−1^) ν_max_: 2242 (nitrile), 1631 (C = C).^1^H NMR (500 MHz, CDCl_3_): *δ* 8.25(s, 1H, triazole), 8.04 (s, 1H, triazole), 7.70-7.69 (d, *J* = 8.5 Hz, 2H, benzonitrile), 7.35-7.33 (d, *J* = 8.5 Hz, 2H, benzonitrile), 7.29-7.26 (m, 1H, phenyl), 7.22 (s, 1H, phenyl), 7.20-7.18 (d, *J* = 7.95 Hz, 1H, phenyl), 6.93 (s, 1H, ethenyl), 6.69-6.68 (d, *J* = 8.2 Hz, 1H, phenyl), ESI-Mass *m/z*: 306 [M]^+^.

#### 4-[2-(4-Chlorophenyl)-1-(1H-1,2,4-triazol-1-yl)ethenyl]benzonitrile (1d)

Yield: 71%, mp 139–146°C. IR (KBr, cm^−1^) ν_max_: 2237 (nitrile), 1630 (C = C).^1^H NMR (500 MHz, CDCl_3_) *δ* 8.25(s, 1H, triazole), 8.04 (s, 1H, triazole), 7.70-7.69 (d, *J* = 8.86 Hz,2H, benzonitrile), 7.34-7.33 (d, *J* = 8.6 Hz, 2H, benzonitrile), 7.27-7.25 (d, *J* = 8.5 Hz, 2H, phenyl),7.24 (s, 1H, ethenyl), 6.80-6.79(d, *J* = 8.5 Hz, 2H, phenyl), ESI-Mass *m/z*: 306 [M]^+^.

#### 4-[2-(2,4-Dichlorophenyl)-1-(1H-1,2,4-triazol-1-yl)ethenyl]benzonitrile (1e)

Yield: 74%, mp 150–153°C. IR (KBr, cm^−1^) ν_max_: 2248 (nitrile), 1629 (C = C).^1^H NMR (500 MHz, CDCl_3_): *δ* 8.16 (s, 1H, triazole), 7.96 (s, 1H, triazole), 7.74-7.72 (d, *J* = 8.35 Hz, 2H, benzonitrile) , 7.42-7.40 (m, 4H, benzonitrile and ethenyl), 7.04-7.01 (m, 1H, phenyl), 6.56-6.55 (m, 1H, phenyl), ESI-Mass *m/z*: 340 [M]^+^.

#### 4-[2-(3-Fluorophenyl)-1-(1H-1,2,4-triazol-1-yl)ethenyl]benzonitrile (1f)

Yield: 74%, mp 145–150°C. IR (KBr, cm^−1^) ν_max_: 2244 (nitrile), 1634 (C = C).^1^H NMR (500 MHz, CDCl_3_): *δ* 8.25(s, 1H, triazole), 8.05 (s, 1H, triazole), 7.71-7.69 (d, *J* = 8.5 Hz,2H, benzonitrile), 7.35-7.33 (d, *J* = 8.5 Hz, 2H, benzonitrile), 7.30-7.26 (m, 1H, phenyl), 7.25 (s, 1H, ethenyl), 7.03-6.99 (m, 1H, phenyl), 6.68-6.66 (d, *J* = 7.75 Hz, 1H, phenyl), 6.58-6.56 (d, *J* = 7.8 Hz, 1H, phenyl), ESI-Mass *m/z*: 290 [M]^+^.

#### 4-[2-(3-Methoxyphenyl)-1-(1H-1,2,4-triazol-1-yl)ethenyl]benzonitrile (1 g)

Yield: 74%, mp 128–132°C. IR (KBr, cm^−1^) ν_max_: 2242 (nitrile), 1631 (C = C).^1^H NMR (500 MHz, CDCl_3_): *δ* 8.24(s, 1H, triazole), 8.05 (s,1H, triazole), 7.69-7.67 (d, *J* = 8.1 Hz, 2H, benzonitrile), 7.34-7.33 (d, *J* = 8.1 Hz, 2H, benzonitrile), 7.27 (s, 1H, ethenyl), 7.21-7.18 (t, *J* = 7.9 Hz, 1H, phenyl), 6.86-6.84 (d, *J* = 7.6 Hz, phenyl), 6.54-6.53 (d, *J* = 7.43 Hz, phenyl), 6.31 (s, 1H, phenyl), 3.66 (s,3H,OMe), ESI-Mass *m/z*: 302 [M]^+^.

#### 4-[2-(4-Methoxyphenyl)-1-(1H-1,2,4-triazol-1-yl)ethenyl]benzonitrile (1 h)

Yield: 74%, mp 140–143°C. IR (KBr, cm^−1^) ν_max_: 2244 (nitrile), 1632 (C = C).^1^H NMR (500 MHz, CDCl_3_) *δ* 8.27(s, 1H, triazole), 8.08 (s,1H, triazole), 7.67-7.65 (d, *J* = 8.7 Hz,2H, benzonitrile) , 7.30-7.29 (d , *J* = 8.7 Hz, 2H, benzonitrile), 7.25 (s, 1H, ethenyl), 6.80-6.78 (d, *J* = 9.2 Hz, 2H, phenyl), 6.77-6.76 (d, *J* = 9.2 Hz, 2H, phenyl), 3.80 (s,3H, OMe), ESI-Mass *m/z*: 302 [M]^+^.

#### 4-[2-(2,4-Dimethoxyphenyl)-1-(1H-1,2,4-triazol-1-yl)ethenyl]benzonitrile (1i)

Yield: 74%, mp 158–161°C. IR (KBr, cm^−1^) ν_max_: 2239 (nitrile), 1632 (C = C). ^1^H NMR (500 MHz, CDCl_3_): *δ* 8.22(s, 1H, triazole), 8.06 (s,1H, triazole), 7.66-7.65 (d, *J* = 8.3 Hz,2H, benzonitrile), 7.50 (s, 1H, ethenyl ), 7.31-7.30 (d, *J* = 8.3 Hz, 2H, benzonitrile), 6.44-6.27 (m, 3H, phenyl), 3.84 (s,3H, OMe), 3.79 (s,3H, OMe), ESI-Mass *m/z*: 332 [M]^+^.

#### 4-[2-(2,3,4-Trimethoxy)-1-(1H-1,2,4-triazol-1-yl)ethenyl]benzonitrile (1j)

Yield: 75%, mp 188–191°C. IR (KBr, cm^−1^) ν_max_: 2246 (nitrile), 1634 (C = C).^1^H NMR (500 MHz, CDCl_3_): *δ* 8.20(s, 1H, triazole), 8.09 (s,1H, triazole), 7.67-7.65 (d, *J* = 8.3 Hz,2H, benzonitrile), 7.51 (s, 1H, ethenyl ), 7.33-7.30 (d, *J* = 8.3 Hz, 2H, benzonitrile), 6.46-6.25 (m, 2H, phenyl), 3.86 (s,3H, OMe), 3.81 (s,3H, OMe), 3.76 (s,3H, OMe), ESI-Mass *m/z*: 3362 [M]^+^.

#### 4-[2-(4-Dimethylamino)-1-(1H-1,2,4-triazol-1-yl)ethenyl]benzonitrile (1 k)

Yield: 72%, mp 158–161°C. IR (KBr, cm^−1^) ν_max_: 2241 (nitrile), 1633 (C = C).^1^H NMR (500 MHz, CDCl_3_) *δ* 8.26(s, 1H, triazole), 8.05 (s,1H, triazole), 7.66-7.65 (d, *J* = 8.5 Hz,2H, benzonitrile), 7.32-7.27 (d, *J* = 8.5 Hz, 2H, benzonitrile), 7.25 (s, 1H, ethenyl), 6.79-6.75 (d, *J* = 9.1 Hz, 2H, phenyl), 6.74-6.72 (d, *J* = 9.1 Hz, 2H, phenyl), 3.79-3.75 (m,6H, Me), ESI-Mass *m/z*: 315 [M]^+^.

### Physicochemical prediction

Marvin was used for chemical drawing, displaying and characterizing chemical structures, calculator plugins were used for structure property prediction and calculation, (version: Marvin 6.0.3, 2013, ChemAxon scientific package, http://www.chemaxon.com).

### Molecular modeling study

Docking studies for selected compounds were performed using Autodock Vina (ver. 1.1.1) [13]. The crystal structure of human placental aromatase cytochrome P450 in complex with androstenedione (code ID: 3EQM, resolution [Å]: 2.90) was retrieved from protein data bank [14-17]. Crystal structure was cleaned from Co-crystallized ligand and water molecules and the protein was converted to pdbqt format using Autodock Tools (1.5.4) [18]. 2Dstructures of ligands converted to 3D in pdbqt format by Openbabel (ver. 2.3.1) [18]. The docking parameters were set on vina docking parameter as follow: center_x = 85.027; center_y = 54.737; center_z = 46.428; size_x =50; size_y =50; size_z =50;. The other parameters were left as default for the program. Finally, the conformation for the best free energy of binding was selected for analyzing the interactions between the macromolecule and selected inhibitors. 3D models of ligand-receptor interactions are generated by using the Autodock Tools (1.5.4) [19].

### Biological assay

#### Cell lines and cell culture

Three human breast cancer cell lines including MDA-MB-231, MCF-7 and T-47D were obtained from National Cell Bank of Iran (NCBI, Iran). The cells were grown in RPMI-1640 medium supplemented with 10% heat-inactivated fetal calf serum (GibcoeBRL, UK), 100 mg/ml streptomycin and 100 U/ml penicillin at 37°C/95% rh/5% CO_2_.

#### In vitro cytotoxicity assay

The in-vitro cytotoxic activity of all synthesized compounds **1a-k** was achieved against three human breast cancer cell lines using MTT colorimetric assay according to the method of Mosman [20]. Cells were seeded in 96-well plates (Nunc, Denmark) and incubated overnight in a humidified air atmosphere at 37°C with 5% CO_2_ to allow cell attachment. The cells were then incubated for another 48 h with various concentrations of compounds **1a-k**. The final concentration of DMSO in the highest concentration of the applied compounds was 1%. In each plate, there were three control wells (cells without test compounds) and three blank wells (the medium with 1% DMSO) for cell viability. Etoposide were used as positive controls for cytotoxicity. After 48 h, the culture medium was removed and 200 μl phenol red-free medium containing MTT (final concentration 0.5 mg/mL) was added to wells, followed by 4 h incubation.

After incubation, the culture medium was then replaced with 100 μl of DMSO and the absorbance of each well was measured by using a microplate reader at 492 nm. For each compound, the concentration causing 50% cell growth inhibition (IC_50_) compared with the control containing 1% DMSO was calculated from concentration response curves by regression analysis.

## Results and discussions

### Chemistry

4-Bromotolunitrile was converted to 4-(4-cyanobenzyl)-1,2,4-triazole and subsequently to corresponding product, 4-[2-aryl-1-(1*H*-1,2,4-triazol-1-yl)ethenyl]benzonitrile (**1a-k**) according to the procedure presented in Scheme 1. Chemical structures, molecular formula and molecular weight of compounds (**1a-k**) are illustrated in Table 1. Reaction yields are presented in chemistry section of methods in this report.

**Table 1:** **Target structures and physicochemical properties**


**No.**	**Comp. Code**	**Ar**	**MW**	**Formula**	**Vander Waals Surface**	**Polar Surface**	**Log P**
**1.**	1a		272	C_17_H_12_N_4_	363.21	54.50	3.12
**2.**	1b		306.75	C_17_H_11_ClN_4_	376.65	54.50	3.72
**3.**	1c		306.75	C_17_H_11_ClN_4_	378.12	54.50	3.72
**4.**	1d		306.75	C_17_H_11_ClN_4_	378.12	54.50	3.72
**5.**	1e		341.19	C_17_H_10_Cl_2_N_4_	393.15	54.50	4.33
**6.**	1f		290.29	C_17_H_11_FN_4_	369.04	54.50	3.22
**7.**	1g		302.33	C_18_H_14_ON_4_	410.22	63.73	2.96
**8.**	1h		302.33	C_18_H_14_ON_4_	409.36	63.73	2.96
**9.**	1i		332.36	C_19_H_16_O_2_N_4_	457.69	72.96	2.80
**10.**	1j		362.38	C_20_H_18_O_3_N_4_	504.76	82.19	2.65
**11.**	1k		315.37	C_19_H_17_N_5_	448.10	57.74	3.23

### Physicochemical prediction

In order to investigate the physicochemical properties of products, Vander Waals surface, polar surface and partition-coefficient (Log P) of compounds (**1a-k**) were predicted by Marvin program and are reported in Table 1. As it shown primary physicochemical criteria were passed by all designed compounds (**1a-k**).

### Cytotoxic activity

The *in vitro* cytotoxic activity of 4[2-aryl-1-(1*H*-1,2,4-triazol-1-yl)ethenyl]benzonitrile (**1a-k**)**,** were tested against three human breast cancer lines including MDA-MB-231, T47D and MCF-7. The various concentrations of the synthetic compounds (final concentration 5, 10, 20, 40, 80 and 100 μg/ml) were applied to calculate IC_50_. The 50% growth inhibitory concentration (IC_50_) for products were calculated and depicted in Table 2.

**Table 2:** ***In vitro*** **cytotoxic activity (IC**_**50**_ **, μg/ml) of compounds 1a-k against breast cancer cell lines** ^a^

**No.**	**Comp. Code**	**Cell lines**		
		**MCF-7**	**MDA-MB-231**	**T-47D**
**1.**	1a	57.1 ± 2.1	87.5 ± 2.5	64.3 ± 1.9
**2.**	1b	63.2 ± 2.6	97.3 ± 3.1	77.1 ± 2.8
**3.**	1c	27.1 ± 1.2	14.5 ± 2.1	76.25 ± 7.0
**4.**	1d	52.3 ± 2.2	43.3 ± 3.4	83.3 ± 5.2
**5.**	1e	78.3 ± 5.7	83.3 ± 7.2	92.3 ± 6.2
**6.**	1f	72.3 ± 5.5	85.3 ± 7.4	87.3 ± 7.5
**7.**	1 g	40.3 ± 2.8	77.4 ± 6.5	69.4 ± 5.7
**8.**	1 h	74.6 ± 6.5	82.3 ± 7.4	14.3 ± 1.1
**9.**	1i	75.3 ± 4.4	89.4 ± 6.1	79.1 ± 7.7
**10.**	1j	69.3 ± 5.3	45.05 ± 6.2	63.3 ± 6.6
**11.**	1 k	55.3 ± 5.1	19.7 ± 1.8	16.8 ± 2.1
**12.**	Etoposide	7.9 ± 0.5	11.1 ± 1.1	8 ± 0.8

^a^The IC_50_ values represent an average of three independent experiments (mean ± SD).

According to MTT assay results in Table 2, 4-[2-(3-chlorophenyl)-1-(1*H*-1,2,4-triazol-1-yl)ethenyl]benzonitrile (**1c**) showed the highest activity against MCF-7 and MDA-MB-231 cell lines with IC_50_ values of 27.1 ± 1.2 and 14.5 ± 2.1 μg/ml, respectively and 4-[2-(4-methoxyphenyl)-1-(1*H*-1,2,4-triazol-1-yl)ethenyl]benzonitrile (**1 h**) exhibited highest activity against T47D cell line with IC_50_ value of 14.3 ± 1.1 μg/ml. As can be seen in Table 2, compound 4-[2-(4-dimethylamino)-1-(1*H*-1,2,4-triazol-1-yl)ethenyl]benzonitrile (**1 k**)**,** showed comparative activity against T47D and MDA-MB-231 cell lines with compound **1 h** and Etoposide withIC_50_ values of 16.8 ± 2.1 and 19.7 ± 1.8 μg/ml, respectively. As it shown in MTT assay results all other synthesized compound did not show good activity against tested cell lines.

### Docking study

**Figure 2:**
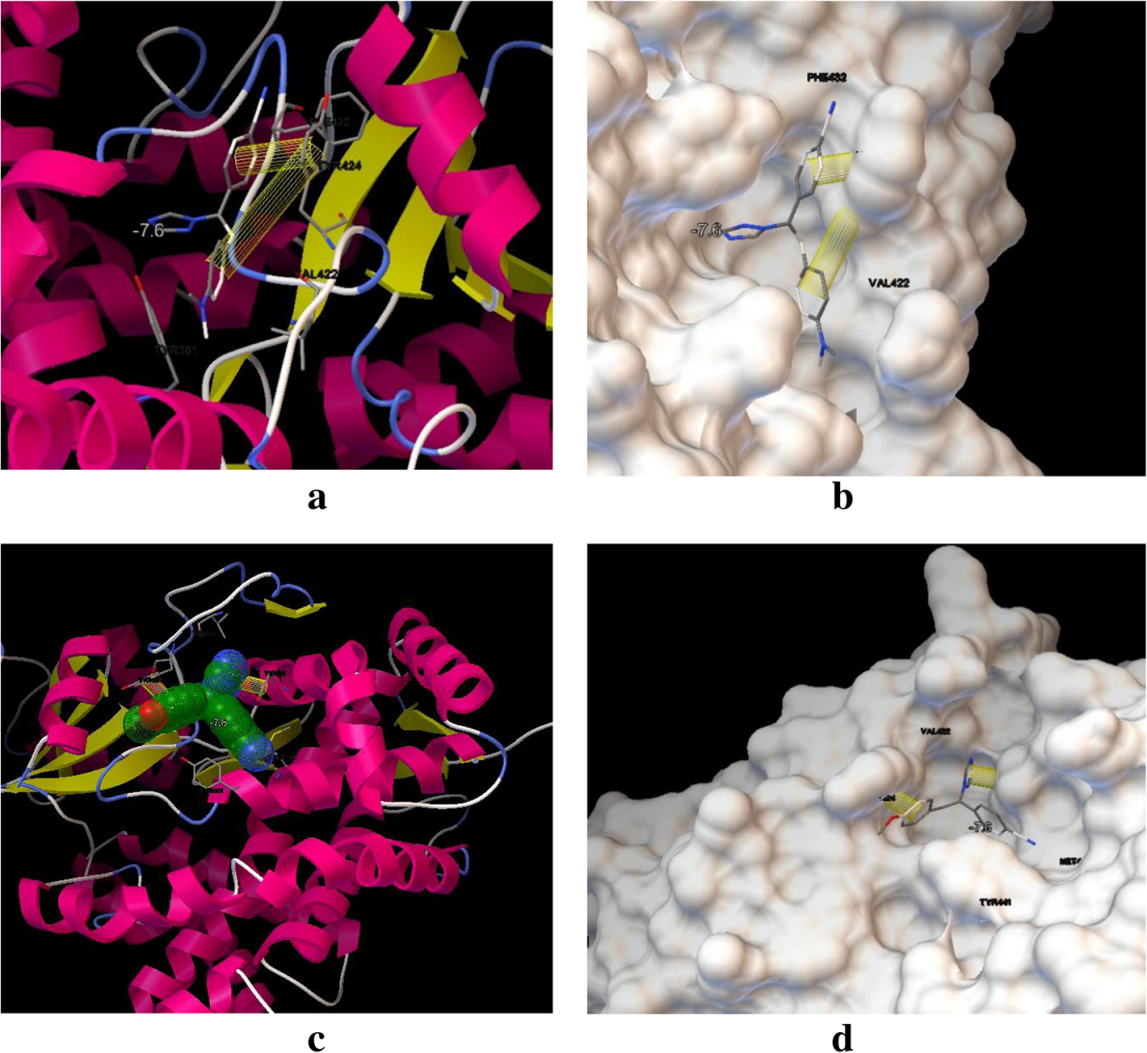
**Presentation of compounds (1c) and (1 k) with aromatase enzyme, π-π interactions showed in yellow cylindrical shape. (a, b** **)** visualization of compound **(** **1c** **)** in enzyme with ribbon and molecular surface views; **(c, d** **)** binding mode of **(** **1 k** **)** in enzyme with ribbon and molecular surface views.

In order to understand the binding mode of active compounds in the active site pocket of aromatase, docking study was performed using Autodock Vina. To attain this aim, the potent compounds, **1c** and **1 k** were docked into target enzyme. Docking strongly suggested that the π-π interaction between adjacent phenyl rings and hydrophobic moieties in enzyme residues –Tyrosine 424 and Tyrosine 361- are effective in activity of biologically active synthesized compounds. According to Figure 2, selected compounds fit in the pocket of aromatase enzyme completely, however missing the potentially hydrogen bond between ligands and macromolecule is responsible for moderate activities of compounds (**1c**) and (**1 k**).

## Conclusion

In the process of anti-cancer drug discovery, to find new potential anti-breast cancer agents, we designed and synthesized a novel series of letrozole analogs. Cytotoxicity evaluation revealed that compounds (**1c**) and (**1 k**) were the most potent compounds with comparative activity with Etoposide. Physicochemical properties of products predicted and the binding mode of (**1c**) and (**1 k**) were predicted by docking simulation; the results revealed that π-π interactions are responsible for the enzyme inhibitions of compounds (**1c**) and (**1 k**).

## References

[1] Dutta U, Pant K (2008). Aromatase inhibitors: past, present and future in breast cancer therapy. Med Oncol.

[2] Vicini F, Beitsch P, Quiet C, Gittleman M, Zannis V, Fine R, Whitworth P, Kuerer H, Haffty B, Keisch M, Lyden M (2011). Five-year analysis of treatment efficacy and cosmesis by the American society of breast surgeons mammo site breast brachytherapy registry trial in patients treated with accelerated partial breast irradiation. Int J Radiant Oncol Biol Phys.

[3] Smith GL, Xu Y, Buchholz TA, Giordano SH, Jiang J, Shih YC, Smith BD (2012). Association between treatment with brachytherapy vs. whole-breast irradiation and subsequent mastectomy, complications , and survival among older women with invasive breast cancer. JAMA.

[4] Cox JA, Swanson TA (2013). Current modalities of accelerated partial breast irradiation. Nat Rev Clin Oncol.

[5] Caporuscio F, Rastelli G, Imbriano C, Delrio A (2011). Structure-based design of potent aromatase inhibitors by high-throughput docking. J Med Chem.

[6] Dowsett M, Cuzick J, Ingle J, Coates A, Forbes J, Bliss J, Buyse M, Baum M, Buzdar A, Colleoni M, Coombes C, Snowdon C, Gnant M, Jakesz R, Kaufmann M, Boccardo F, Godwin J, Davies C, Peto R (2010). Meta-analysis of breast cancer outcomes in adjuvant trials of aromatase inhibitors versus tamoxifen. J Clin Oncol.

[7] Zonouzi A, Mirzazadeh R, Safavi M, Kabudanian Ardestani S, Emami S, Foroumadi A (2013). 2-Amino-4-(nitroalkyl)-4H-chromene-3-carbonitriles as New Cytotoxic Agents. Iran J Pharm Res.

[8] Vosooghi M, Yahyavi H, Divsalar K, Shamsa H, Kheirollahi A, Safavi M, Ardestani SK, Sadeghi-Neshat S, Mohammadhosseini N, Edraki N, Khoshneviszadeh M, Shafiee A, Foroumadi A (2013). Synthesis and In vitro cytotoxic activity evaluation of (*E*)-16-(substituted benzylidene) derivatives of dehydroepiandrosterone. Daru J Pharm Sci.

[9] Ketabforoosh S H M E, Kheirollahi A, Safavi M, Esmati N, Ardestani S K, Emami S, Firoozpour L, Shafiee A, A Foroumadi A: **Synthesis and anti-cancer activity evaluation of new dimethoxylated chalcone and flavanone analogs.** *Arch. Pharm. (Weinheim)* 2014, in press: doi:10.1002/ardp.201400215. 10.1002/ardp.201400215 25201534

[10] Noushini S, Alipour E, Emami S, Safavi M, Ardestani SK, Gohari AR, Shafiee A, Foroumadi A (2013). Synthesis and cytotoxic properties of novel (*E*)-3-benzylidene-7-methoxychroman-4-one derivatives. Daru J Pharm Sci.

[11] Alipour E, Mousavi Z, Safaei Z, Pordeli M, Safavi M, Firoozpour L, Mohammadhosseini N, Saeedi M, Ardestani SK, Shafiee A, Foroumadi A (2014). Synthesis and cytotoxic evaluation of some new[1,3]dioxolo[4,5-g]chromen-8-one derivatives. Daru J Pharm Sci.

[12] Doiron J, Soultan AH, Richard R, Touré MM, Picot N, Richard R, Cuperlović-Culf M, Robichaud GA, Touaibia M (2011). Synthesis and structure-activity relationship of 1- and 2-substituted-1,2,3-triazole letrozole-based analogues as aromatase inhibitors. Eur J Med Chem.

[13] Trott O, Olson AG (2010). Software news and update AutoDockVina: improving the speed and accuracy of docking with a new scoring function, efficient optimization, and multithreading. J Comput Chem.

[14] Ghosh D, Griswold J, Erman M, Pangborn W (2009). Structural basis for androgen specificity and oestrogen synthesis in human aromatase. Nature.

[15] Suvannang N, Nantasenamat C, Isarankura-Na-Ayudhya C, Prachayasittikul V (2011). Molecular docking of aromatase inhibitors. Molecules.

[16] Thakur A, Timiri AK (2014). Designing of potential new aromatase inhibitor for estrogen dependent disease: a computational approach. World J Pharm Sci.

[17] Mirzaie S, Chupani L, Barzegari Asadabadi E, Shahverdi AR, Jamalan M (2013). Novel inhibitor discovery against aromatase through virtual screening and molecular dynamic simulation: a computational approach in drug design. EXCLI J.

[18] Sanner MF (1999). Python: a programming language for software integration and development. J Mol Graph Mod.

[19] O'Boyle NM, Banck M, James CA, Morley C, Vandermeersch T, Hutchison GR (2011). Open babel: an open chemical toolbox. J Cheminform.

[20] Mosmann T (1983). Rapid colorimetric assay for cellular growth and survival: application to proliferation and cytotoxicity assays. J Immunol Methods.

